# China’s LNG import risk assessment based on the perspective of global governance

**DOI:** 10.1038/s41598-022-20090-1

**Published:** 2022-09-21

**Authors:** Weijing Zeng, Xianfeng Zhang, Na Li, Xiaobo Wang, Xiaolin Wang

**Affiliations:** 1 School of Economics and Management, China University of Geosciences (Wuhan), Wuhan, 430074 China; 2 Research Center of Resource and Environmental Economics, China University of Geosciences (Wuhan), Wuhan, 430074 China

**Keywords:** Environmental social sciences, Energy science and technology

## Abstract

The uncertainty of LNG import risks will have a significant impact on China's energy security. This paper establishes a multi-agent game model based on the current LNG transportation network and global LNG supply and demand pattern, evaluates the LNG import risks faced by China under the global governance model, and simulates and predicts the optimal LNG import strategy of China in 2030. The research results show that between 2007 and 2020, China’s LNG import risks increased rapidly compared with political risks and national risks. From the perspective of risk identification, the comprehensive risk of China's imports from Southeast Asia and Australia is lower. However, due to the increasing demand gap for LNG, the Middle East, and Africa are still necessary supply sources. It is estimated that by 2030, the future LNG market will be oversupplied, and the United States is expected to become the world's top LNG supplier. China will reduce its dependence on Africa and the Middle East, and import a large amount of natural gas from the United States, Australia, Qatar, and Russia to reduce supply risks. From the perspective of import market competition, the new analysis model proposed in this article provides an effective tool for exploring the optimal strategy for LNG import.

## Introduction

Liquefied natural gas (LNG), as efficient and clean energy, plays an important role in the global energy trade pattern with its flexible advantages of safe storage and easy transportation and has gradually become an important choice for energy strategies of various countries^1^. From 2008 to 2020, the LNG trade increased rapidly from 235 billion cubic meters (bcm) to 487.9 bcm^2^. In 2017, China's LNG imports were second only to Japan, becoming the world's second-largest LNG importer. The continuous improvement of LNG trade activity has led to increasingly fierce competition between importing countries and exporting countries, and the trade pattern may be reshaped^3^. The performance is as follows: firstly, the influence of the LNG buyer's market under the oversupply situation of the global natural gas market^4^; the second is the competition between emerging LNG exporting countries (such as the United States) and traditional exporting countries (such as Qatar and Australia)^5^, and LNG exports (such as the United States) and the game of pipeline natural gas exports (Russia)^6^. In addition, affected by factors such as price fluctuations, transportation interruptions, and geopolitics, the global LNG trade will face a higher risk of uncertainty, threatening the supply security of LNG importing countries^7^.

As the world’s second-largest LNG importer, China imported 94 bcm of LNG in 2020, accounting for 51.8% of the total global natural gas imports^2^, and has become an important part of global LNG imports. However, statistics show that the rapid increase in LNG imports has led to China’s external dependence on LNG from 43% in 2020 to 62% in 2030^2^, which seriously affects the security of China’s natural gas supply and promotes the redistribution of global LNG import and export trade pattern. Therefore, how to reduce the risk of China's LNG import is a necessary practice that urgently needs to be resolved to ensure the stability of China's natural gas market, and it is also an important exploration in global LNG trade negotiations.

## Literature review

With the increasing activity of LNG trade, the risk and prevention of LNG import have aroused widespread concern. Based on a summary of existing research, LNG imports face three main risks, namely, economic risks due to natural gas prices and import costs, political risks due to political turbulence in exporting countries, and maritime transportation risks^8–10^. Based on risk identification, a large number of studies have focused on LNG import risk assessment methods. Scholars such as Tixier et al.^11^ summarized the risk assessment method of the dynamic system and summarized the risk assessment process into hazard identification, risk assessment, and risk stratification. Vivoda et al.^12^ respectively used principal component analysis and Herfindahl–Hirschman Index (HHI) to evaluate the impact of natural gas supply interruption on major Asian gas importing countries and the diversified investment portfolio of LNG imports. The results show that China is the second most risky country for LNG imports in Asia, but HHI has improved. Scholars such as Kong et al.^13^ and Biresselioglu^9^ transformed the LNG import risk problem into a linear or mixed-integer programming model to explore the optimal import risk combination, to reduce the impact of LNG supply interruption caused by changes in the political environment of the supplier country, pirate attacks and strait closure. Some scholars have also tried to construct a network model of the influencing factors of LNG import risks, and analyze the regional combination strategies to deal with LNG supply risks^14^. For example, Huang Tao^15^ used the fuzzy network method to assess the risk of China's LNG route. In addition, Magnier et al.^16^ adopted a simplified coarse-grained model to select the LNG import trade portfolio strategy in Europe and Asia–Pacific markets.

The above studies provide the basis for LNG import risk evaluation and strategic analysis. However, these studies aim to judge the optimal export source of LNG through indicators but fail to fully consider the joint effect of various risks on the choice of import channels and the inherent power of LNG import evolution. The subjective viewpoint-oriented weighting system used by many researchers may distort indicator weights and bias the evaluation results. Few studies have taken global LNG trade as the background and described it as a game system composed of supply and demand sides. It is difficult to give changes in import and export strategies. Mixed complementarity programming (MCP) provides a general mathematical framework for the equilibrium modeling of energy markets^17,18^. The main idea is to describe each participant as solving a separate optimization problem and simulate the market performance generated by the game behavior of market players. Many researchers have used this method to conduct simulation research on the natural gas market, such as the research on the security of supply and market power in Europe^19^ and the research on third-party access systems in North America^20^. However, few researchers use this method to study LNG import risk. Multi-agent game model based on MCP can bring import and export countries into a game system, which can not only quantify import risks but also give optimal import strategies under different market Settings. The advantages mentioned above make up for the deficiency of existing research to some extent.

## Overview of LNG import multi-subject market

As of the end of 2020, there were 17 major LNG exporting countries and 40 importing countries^2^ (The related information is shown in Table 1), ignoring a few minor countries. According to the British Petroleum (BP) report, the total export volume of 17 LNG exporting countries accounted for 98.23% of the total global LNG export volume. LNG exporting countries are mainly Qatar, Australia, Malaysia, the United States, Nigeria, Russia, Indonesia, and so on. The International Energy Agency (IEA) forecasts that the US could become the world's largest LNG exporter by 2024^21^. The commissioning of the Yamal LNG project in the Russian Arctic and the opening of the Arctic shipping route will also enhance Russia's LNG export capacity^5^. The LNG import area can be divided into the North American market, Central and South American market, European market, and Asian market according to the import area. Among them, the Asian market is currently the largest demand area for LNG, with imports accounting for more than 70%. Asian countries such as China, Japan, South Korea, and India accounted for 19.27%, 20.91%, 11.33%, and 7.3% of LNG imports in 2020. To highlight China's status, this paper discusses China as an independent market. Because Japan and South Korea are geographically close and share a high share of imports, the Japanese and South Korean markets are discussed as a whole. Similarly, India and the remaining Asian and African countries are located in South Asia. This article divides them into the Rest of Asia and Africa market for exploration. Europe is the world's second-largest LNG import region, accounting for 23.53% of the global total. LNG imports in the Americas only account for less than 5% of the world's total, but they have basically achieved self-sufficiency.

**Table 1:** Information on the six major import markets in 2020.

Importing markets	Market shares (%)	Countries included	LNG import sources
China	19.27	China	Algeria, Nigeria, Qatar, USA, Eygpt, Angola, Peru, Australia, Russia, Malaysia, T&T, PNG, UAE, Indonesia, Oman, Brunei
Japan and South Korea	32.25	Japan, South Kore	Nigeria, Qatar, USA, Eygpt, Angola, Peru, Australia, Russia, Malaysia, T&T, PNG, UAE, Indonesia, Oman, Brunei
Rest of Asia and Africa	21.2	India, Malaysia, Pakistan, Singapore, Thailand, Other Asia Pacific, Kuwait, UAE, Other Middle East &Africa	Algeria, Norway, Nigeria, Qatar, USA, Eygpt, Angola, Australia, Russia, Malaysia, T&T, PNG, UAE, Indonesia, Oman, Brunei
Europe	23.53	Belgium, France, Italy, Spain, Turkey, UK, Other EU	Algeria, Norway, Nigeria, Qatar, USA, Eygpt, Angola, Peru, Russia, T&T
North America	0.9	Canada, Mexico, US	Norway, Nigeria, USA, Peru, Australia, T&T, Indonesia
Central and South America	2.85	Argentina, Brazil Brazil, Chile, Other S&Cent. America	Algeria, Norway, Qatar, USA, Angola, Australia, Russia, T&T

In these six import markets, The North American market and the Central and South American markets share a small share of LNG import demand, and the import sources are relatively overlapping, which basically comes from intra-regional imports. The market shares of LNG imports in Central and South America are higher than that in North America. In addition to the American countries, it also has trade relations with North Africa and Qatar. The European market has a high demand for LNG and low diversification of imports, mostly from the Americas, the Middle East, North Africa, and many other countries. In addition, to get rid of the dependence on Russian pipeline natural gas and the requirements of low-carbon policies, European LNG foreign dependence will continue to increase in the future^22^. The United States may become one of the main sources of LNG imports in Europe, which will also intensify the competition with other import markets for LNG. The LNG import maturity of China, Japan and South Korea, and India is relatively high. In terms of import sources, the three markets share many of the same import sources and have trade relations with America, North Africa, the Middle East, and especially Southeast Asia. These three markets account for the share of Southeast Asian LNG exports. With India's economy growing rapidly and South Korea still using LNG as an alternative to coal, gas consumption in both countries will continue to grow in the future^23,24^. However, Japan's demand for LNG will not increase significantly in the future as the proportion of renewable energy increases.

China began to import LNG in 2007. As the growth rate of domestic supply is lower than that of natural gas consumption, a large part of the demand gap needs to be met by LNG imports, mainly from Australia, Qatar, Malaysia, and Indonesia. However, many risk factors will affect the import process, such as the closure of the Straits of Hormuz and Malacca passage, the stability of the political pattern in the Middle East, China's relations with the U.S. and so on. By 2020, China's dependence on foreign natural gas will have reached 43%. In recent years, driven by the energy transition, the Chinese government has vigorously implemented the policy of replacing coal with gas, while at the same time, considering environmental factors, China has proposed the goal of achieving carbon neutrality by 2060. This will drive the continued growth of China's LNG imports. Under the background of China's diversified import strategy, it is bound to compete with big natural gas importers in the future. How to reduce the risk of LNG import and ensure the security of supply, China is facing many opportunities and challenges.

## Models, methods, and data

### LNG import multi-agent market game model

1. Global LNG import and export market structure

The multi-agent market game model is formulated as an MCP, a more general modeling approach than, e.g., linear programming (LP) or quadratic programming (QP), and a generalization of pure nonlinear complementarity problems (NCP)^25^. In energy markets, mixed complementarity problems are increasingly used, which is mainly realized through the Karush–Kuhn–Tucker (KKT) conditions and market clearing conditions. According to the principle of the complementarity problem^26^, the model constructed in this paper takes each importing country as a separate topic to optimize its objective function and lists the constraints and dual variables that each subject needs to satisfy. The objective function of each import market is to minimize import risk costs. The import risk cost considered in this paper mainly includes the import price cost of LNG, the country risk cost of the import source country in the transaction process, and the sea transportation risk cost in the transportation process. Restrictions on import demand and export supply, import and export diversification, and shipping channel capacity are related constraints.

**Figure 1:**
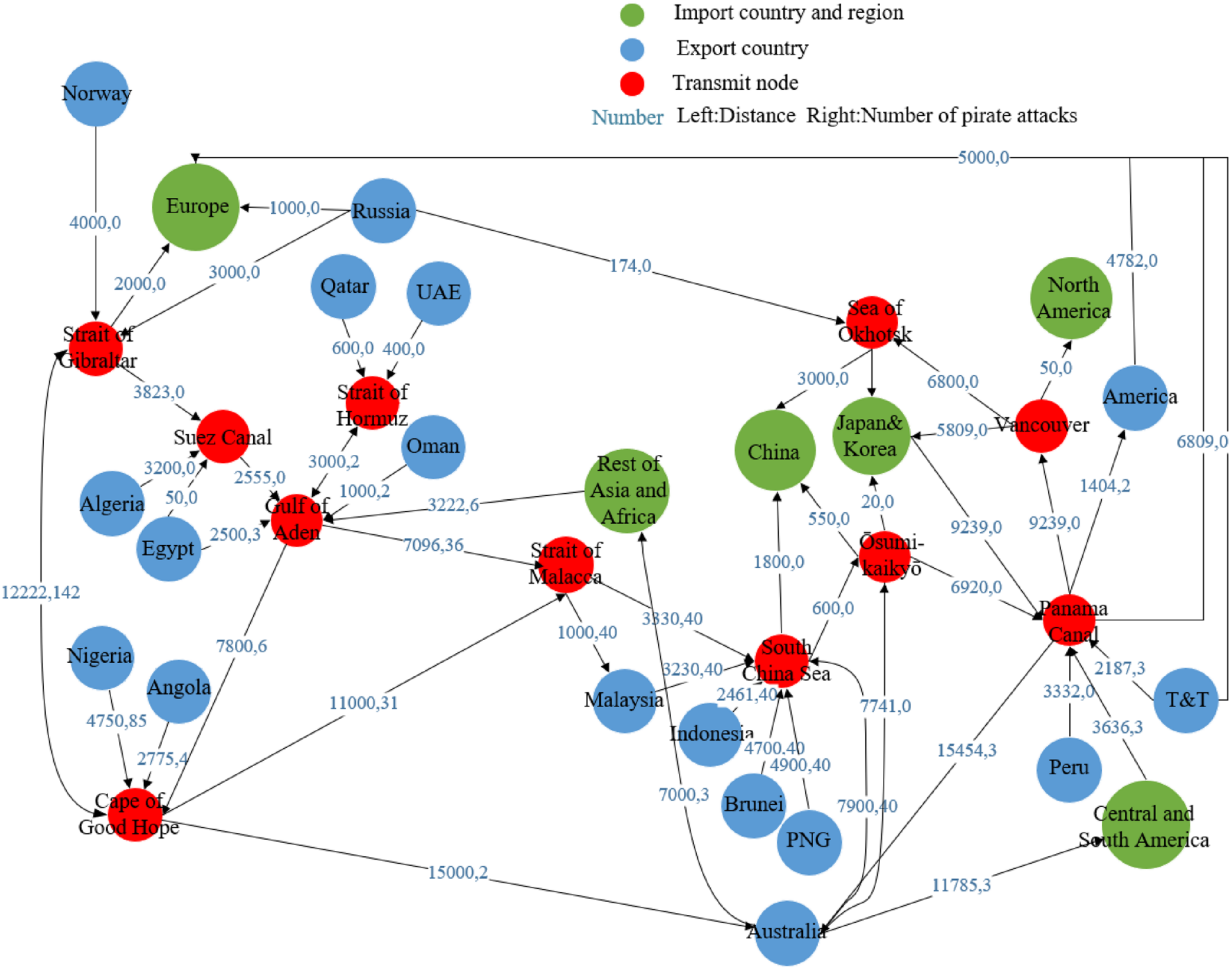
Generalized map of global LNG trade shipping routes (The unit of distance is a kilometer).

The global LNG trade and transportation network is a transportation network composed of three layers of nodes: exporting countries, transshipment channels, and LNG import markets. This article builds a generalized network of global LNG shipping routes (Fig. 1) to show the transaction relationship between each LNG import market and exporting country and the transportation relationship from each channel. According to the current status of global LNG trade in 2020, this paper sets up 17 LNG export nodes (blue solid circles in Fig. 1) and 6 LNG import nodes (green solid circles in Fig. 1) to indicate exporting countries and import markets. In addition, we have set up 11 transit nodes (red solid circles in Fig. 1) for the Panama Canal, The Strait of Gibraltar, the Strait of Malacca, the Strait of Hormuz, the Sea of Okhotsk, and the Cape of Good Hope, which are the main routes of the LNG route^27^.

2. Objective function

Table 2 for the explanation of the variables involved in this model.

**Table 2:** List of model parameters and their meanings.

Parameter declaration	Explanation	Parameter declaration	Explanation
**Sets**		$wt_{p}$	Each LNG-demanding country faces the political risk weight of the exporting country;
$s \in S$	LNG exporting country, *S* is the set of exporting countries	$wg_{p}$	Each LNG demand country adopts a specific transportation route risk weight;
$p \in P$	LNG importing country, *P* is the collection of importing countries	**Decision variables**	
$n,m \in A$	n, m are the nodes of the global LNG trade maritime network, and A is the maritime network collection	$flow_{pnm(p)}$	The volume of LNG transported by the importing country p via the shipping route *(n(p), m)*;
$n(s) \in A$	*n(s)* is the maritime network node where the LNG exporting country *S* is located	$flow_{pnm}$	The volume of LNG transported by the importing country p via the sea route *(n, m)*;
$m(p) \in A$	*m(p)* is the maritime network node where LNG importing country *P* is located	$flow_{pn(s)m}$	The volume of LNG transported by the importing country p via the shipping route *(n(s), m)*;
**Parameters**		$trade_{sp}$	LNG trade volume between exporting country s and importing country *P*;
$Supply(s)$	The largest LNG export volume of the exporting country *S*, unit:10^8^ m^3^/a;	**Dual variables**	
$Demand\left( p \right)$	The minimum LNG import volume of the importing country *P*, unit:10^8^ m^3^/a;	$\lambda_{s}$	The scarce rent for the largest supply in the exporting country *S* ;
$Capacity_{nm}$	The transportation capacity on the route *(n, m)*, unit:10^8^ m^3^/a;	$\lambda_{p}$	The scarcity rent for the smallest import volume of importing country *P*;
$TR_{pnm}$	The transportation risk that the importing country *P* faces through the sea route *(n, m)*, unit: dimensionless;	$\beta_{sp}^{\min }$	The scarcity rent of the smallest supply from exporting country *S* to importing country *P*;
$Price_{sp}$	LNG transaction price between exporting country *s* and importing country *P*, unit: dollars/m^3^;	$\beta_{sp}^{\max }$	The scarcity rent is bound by the maximum import volume of importing country *P* from exporting country *S* ;
$GR_{sp}$	The political risk faced by exporting country *s*, unit: dimensionless;	$\gamma_{n(s)}$	The shadow price of exporting country traffic clearing at network node *n(s)*;
$Scale_{sp}^{\min }$	Exporting country *S* exports the smallest proportion of importing country *P*, to ensure the diversification of exports. Unit:10^8^ m^3^/a;	$\gamma_{n(p)}$	The shadow price of the traffic cleared at the network node *n(p)* in the importing country *P*;
$Scale_{ps}^{\max }$	Importing country *P* has the largest proportion of imports from exporting country *S*, thus ensuring the diversification of imports. Unit:10^8^ m^3^/a	$\gamma_{m}$	The shadow price of clearing traffic at the shipping network node *m*;
$we_{p}$	Import cost weight of each LNG demand country;	$\delta_{nm}$	The scarce rent for the maximum transportation capacity of the sea route *(n, m)*;

**Price risk.** Import price cost refers to the actual price of LNG paid during the import process, including liquefaction cost and freight cost. Since liquefaction cost has nothing to do with the purchase policy, this item is excluded from the calculation. In addition, during transportation, it is assumed that the unit freight cost is the same, this article obtains the transaction prices between LNG importing countries and exporting countries from the IEA. The LNG import market hopes to minimize the cost of LNG import during the import process, so the objective function in Eq. (1):

1 $$\it {\text{min Z}}_{1p} = \sum\limits_{s} {trade_{sp} } \times price_{sp}$$

*Z*_*1p*_ represents the total cost of importing prices from each exporting country *s* in each importing country market *p*, and *trade*_*sp*_ is the transaction volume between the exporting country *s* and the importing country *p*.

**Country risk of the import source country.** Country risk is a risk related to the exporting country. This risk is affected by many determinants, such as the political climate and economic stability of the exporting country. This article uses the International Country Risk Guide (ICRG) indicator proposed by the Political Risk Services Group (PRS) to quantify the risk of LNG exporting countries. This rating reflects various aspects of the country’s political risks, such as government stability, socio-economic conditions, and internal or external conflicts^9^. The country risk *GR*_*sp*_ indicator is shown in Eq. (2).

2 $$GR_{sp}^{{}} = (100 - ICRG_{s * }^{{}} )/100$$

In the imported LNG, the goal of each import market for the country risk of the import source country is to minimize the import political risk, so the objective function is as follows:

3 $$\it {\text{min Z}}_{2p} = \sum\limits_{s} {Trade_{sp} } \times GR_{sp}$$

*Z*_*2p*_ represents the total country risk of the import source country in each importing market *p*.

**Transportation risk.** The import market transportation risk refers to the shipping risk of LNG transported from the exporting country to the importing country via a specific route, which is affected by factors such as transportation distance and pirate attacks^16^. The transportation distance mainly affects the cost risks related to the price of liquefied natural gas, such as fuel, steamship, and insurance. The greater the distance, the higher the risk. During transportation, it will pass through some key passages such as the Strait of Malacca. Pirate attacks are an important factor affecting the safety of LNG transportation. In this paper, the transportation distance and the number of pirate attacks are combined as the transportation risk. *TR*_*pnm*_ is the transportation risk that the importing country *p* faces through shipping routes *(n, m)*, and the index quantification process is as shown in Eq. (4):

4 $$TR_{pnm} { = }\frac{{D_{nm} }}{{D_{{_{nm} }}^{\max } }} + \frac{{Risk_{nm} - Risk_{{_{nm} }}^{\min } }}{{Risk_{{_{nm} }}^{\max } - Risk_{{_{nm} }}^{\min } }}$$

*D*_*nm*_ represents the transportation distance between maritime routes *(n, m)*, and *Risk*_*nm*_ represents the number of pirate attacks on maritime routes *(n, m)*.

Each import market minimizes the expected transportation risk during the transportation process, and the objective function is as shown in Eq. (5):

5 $$\it {\text{min Z}}_{3p} = \sum\limits_{(m,n) \in A} {flow_{pnm} } \times TR_{pnm}$$

*Z*_*3p*_ represents the total transportation risks faced by each import market *p* on route *(n, m)*, and *flow*_*pnm*_ is the traffic volume of the importing country *p* through the route *(n, m)*.

**Total import risk.** Different LNG import markets are expected to minimize the import risk when importing LNG. Although reducing the import price cost, import source country risk, and import market transportation risk are common factors for all LNG import markets to minimize risk, risk aversion policies are different in each import market. After normalizing different indicators, the three import targets are incorporated into the total import risk of each LNG import market by using different weights. As Eq. (6):

6 $$\it {\text{min Z}}_{p} = we_{p} \times \sum\limits_{s} {trade_{sp} } \times price_{sp} + wt_{p} \times \sum\limits_{(m,n) \in A} {flow_{{{\text{p}}nm}} } \times TR_{pnm} + wg_{p} \times \sum\limits_{s} {Trade_{sp} } \times GR_{sp}$$

Among them, *Z*_*p*_ represents the total import risk of the LNG import market *p*. *we*_*p*_, *wt*_*p*_, and *wg*_*p*_ are respectively the weights of import price risk, transportation risk, and country risk in the import market *p*.

3. Constraints

LNG import faces many restrictions. This article examines the import market clearance conditions, import and export diversification constraints, transportation channel flow clearance conditions, and transportation capacity constraints. The establishment process is as follows:

7 $$- \sum\limits_{p} {trade_{sp} + Supply_{s} \ge } 0\quad (\lambda_{s} \ge {0})\quad \forall s$$

8 $$\sum\limits_{s} {trade_{sp} } - Demand_{p} \ge {0}\quad (\lambda_{p} \ge 0)\quad \forall p$$

Constraint (7) means that for exporting countries, the sum of exports of each LNG exporting country to each LNG import market shall not exceed its maximum supply. Constraint (8) means that for each LNG import market, the total import volume of each LNG import market from each exporting country shall not exceed its LNG demand. The Greek letters in the parentheses of Eq. (7) and Eq. (8) represent the dual variable (lagrangian multiplier) of the scarce trade volume.

To ensure the diversification of imports and exports of LNG trading countries and prevent energy supply problems caused by single import and export, it is necessary to set constraints on diversified exports and diversified imports.

9 $$- Scale_{sp}^{\min } \cdot Supply_{s} + trade_{sp} \ge {0}\quad (\beta_{sp}^{\min } \ge 0)\quad \forall s,p$$

10 $$Scale_{sp}^{\max } \cdot Demand_{p} - trade_{sp} \ge {0}\quad (\beta_{sp}^{\max } \ge 0)\quad \forall s,p$$

Constraint (9) is the condition of export diversification of exporting countries, and the export volume of each exporting country to a single LNG import market should not be less than its minimum export volume. $Scale_{sp}^{\min }$ is the minimum proportion of exporting countries *s* to the import market *p*. Constraint (10) is the diversification of imports in the LNG import market, and the import volume of each import market from a single exporting country should not be higher than its maximum import volume. $Scale_{sp}^{\max }$ is the highest proportion of imports from the import market *p* to the exporting country *s*. The Greek letters in the parentheses of Eq. (9) and Eq. (10) represent the dual variable of the constraints of import and export diversification and the safety cost of export diversification and import diversification.

It is necessary to connect the transaction volume with the transportation channel flow in the import market to establish the market clearing conditions of the transportation network, that is, the network flow transfer equation, as in Eqs. (11), (12), (13). The corresponding dual variable is the shadow price of the clearing of each transit node.

11 $$\sum\limits_{m \ne n} {flow_{pn(s)m} } - trade_{sp} = {0}\quad (\gamma_{n(s)} \;\;free)\quad \forall n(s),p$$

12 $$\sum\limits_{n \ne m} {flow_{pnm(p)} } - trade_{sp} = {0}\quad (\gamma_{m(p)} \;\;free)\quad \forall m(p),p$$

13 $$\sum\limits_{m \ne n} {flow_{pmn} } - \sum\limits_{n \ne m} {flow_{pnm} } = 0\quad (\gamma_{pn} \;\;free)\quad \forall p,n$$

14 $$- \sum\limits_{p} {flow_{pnm} } + Capacity_{nm} \ge 0\quad (\delta_{nm} \ge 0)\quad \forall n,m$$

The constraint condition (13) is the flow balance equation between the transfer nodes: the total amount flowing through a certain transfer node is equal to the total amount flowing out of the node. Constraint (14) is the transportation volume restriction condition of the transportation channel: the sum of the flow of all the import markets through the transportation channel is not higher than the maximum carrying capacity of this channel, and the corresponding dual variable is the maximum transportation capacity of the shipping route *(n, m)* scarce rent.

4. Solution method for model

This part uses the import risk cost minimization principle, deduces the KKT condition from the first-order condition of each decision variable, and combines it with the market clearing condition, which is the key to solve the LNG multi-agent market game mode. And then we use GAMS software to solve the model established above and simulate the natural gas import market equilibrium under different conditions. KKT conditions and market clearing conditions are no longer listed one by one.

### Data source and processing

The data of the largest LNG export volume *Supply(s)* of the world's major LNG exporting countries and the smallest LNG import volume *Demand(p)* of the import market used in this article are from BP (2021). The trade price *Price*_*sp*_ of each LNG import market *p* and exporting country *s* are calculated by trade data, and the data comes from IEA (2021). The maximum export ratio of an exporting country to a certain country is the ratio of the exporting country's maximum export to a certain country to the export volume, calculated based on BP (2021). The minimum proportion of a country's imports from a country in demand is the ratio of the minimum amount of imports from a country to the total amount of imports in the import market, calculated based on BP (2021). The national political risk *GRsp* uses the ICRG risk index data released by PRS. The LNG capacity data of each channel’s Capacity comes from the International Maritime Organization (IMO) (2021). The sea distance data between the nodes of each network channel is obtained through the online map measurement tool. The data on the number of piracy attacks between nodes comes from the International Chamber of Commerce (ICC) (2021)^28^.

## Result

Our results are divided into two main parts. Firstly, we use the model to evaluate the import risk of global historical years, and select 2007,2015, and 2020 for analysis. Second, we simulated the future risks and strategies based on forecasts for global LNG trade in 2030.

### Global import risk evaluation

In 2007, China started importing natural gas for the first time. In 2015, China's dependence on foreign gas exceeded 30%. As of 2020, China's dependence on foreign countries has reached 43%, making China the world's second-largest LNG importer. Since the dependence on imports exceeds 30%, it will become an important factor affecting energy security^29^, so we have chosen these three years to verify and analyze the risks of China and other major importing markets in the LNG global governance system.

**Figure 2:**
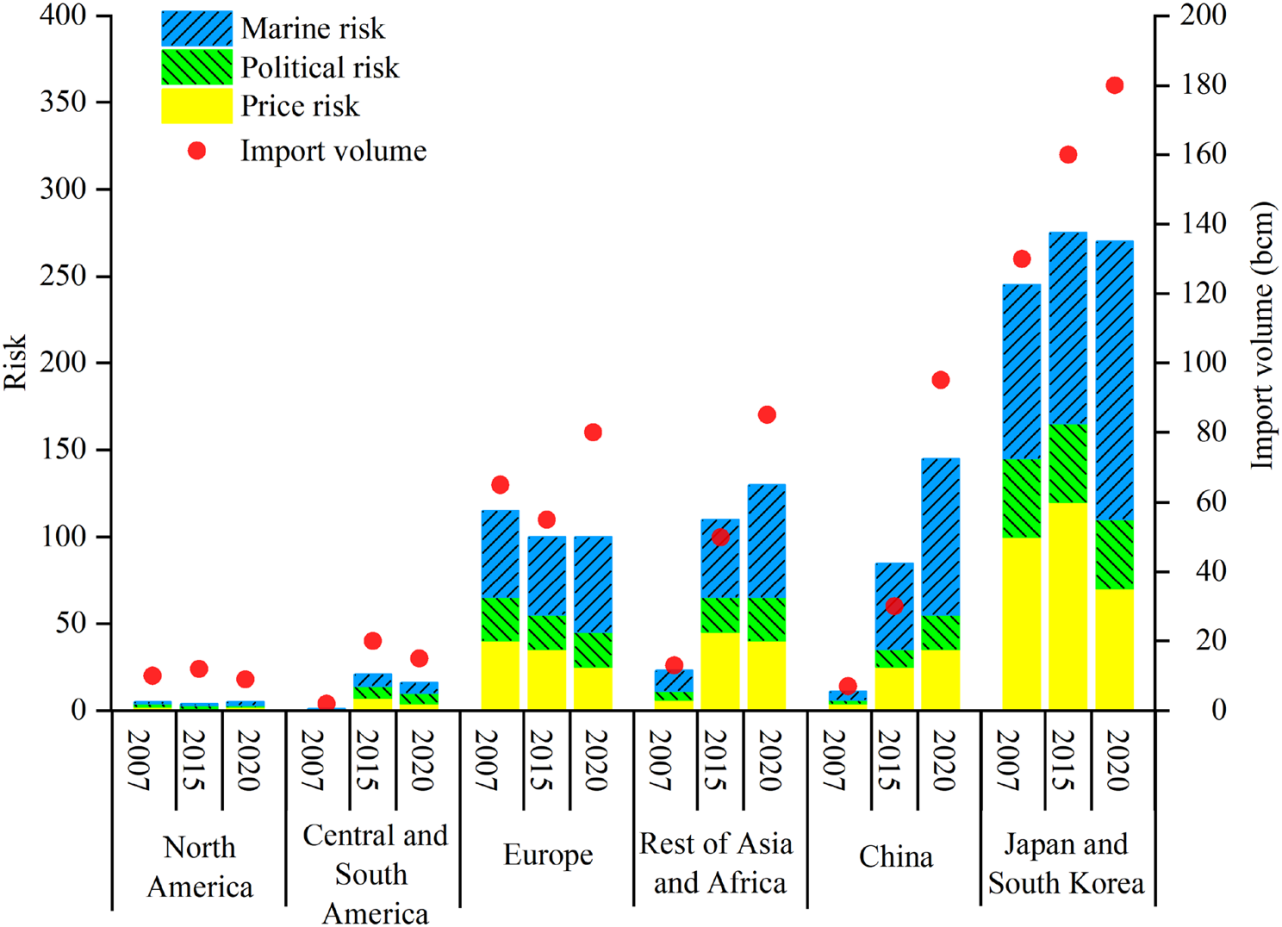
The comparison of LNG import risks in different import markets in different years.

Figure 2 shows the comparison of LNG import risks in different import markets in different years. In general, China’s import risks are showing an increasing trend year by year. The most important factor is the increase in demand. We found that in this transition process, the impact of risk is also different. Maritime risk is the risk with the greatest impact. Although the maritime risk for imports in 2007 is limited to the Strait of Malacca, it accounts for the highest proportion of the three source risks. After 2015, the increase in LNG import risks comes from imports from the Middle East and Africa, and the transportation risks in the Straits of Malacca and Hormuz have become the most important factors^30^. Due to the high similarity of import sources between the Japanese and South Korean market and the Chinese market, the above risk analysis is also suitable for analyzing the shipping risks of LNG imports in Japan and South Korea.

However, different market risks also have differences in price risks. We found that Japan and South Korea’s price risk has a downward trend from 2015 to 2020. The reason behind this is due to the transformation of the Asia–Pacific region’s purchase price system: the transition from the oil–gas pegged era to the gas-to-gas pricing model is similar to the decoupling of oil and gas in North American and European markets^31^. In fact, after 2018, the world LNG supply and demand pattern gradually changed from a shortage of supply to a trend of oversupply. Relying on the spot pricing method, the export competition to the Asia–Pacific market is gradually increasing, the price is lowered, and the price risk is also reduced. However, under the huge LNG import demand, the long-term import contract transaction method is still adopted, and the price is still the long-term contract price.

**Figure 3:**
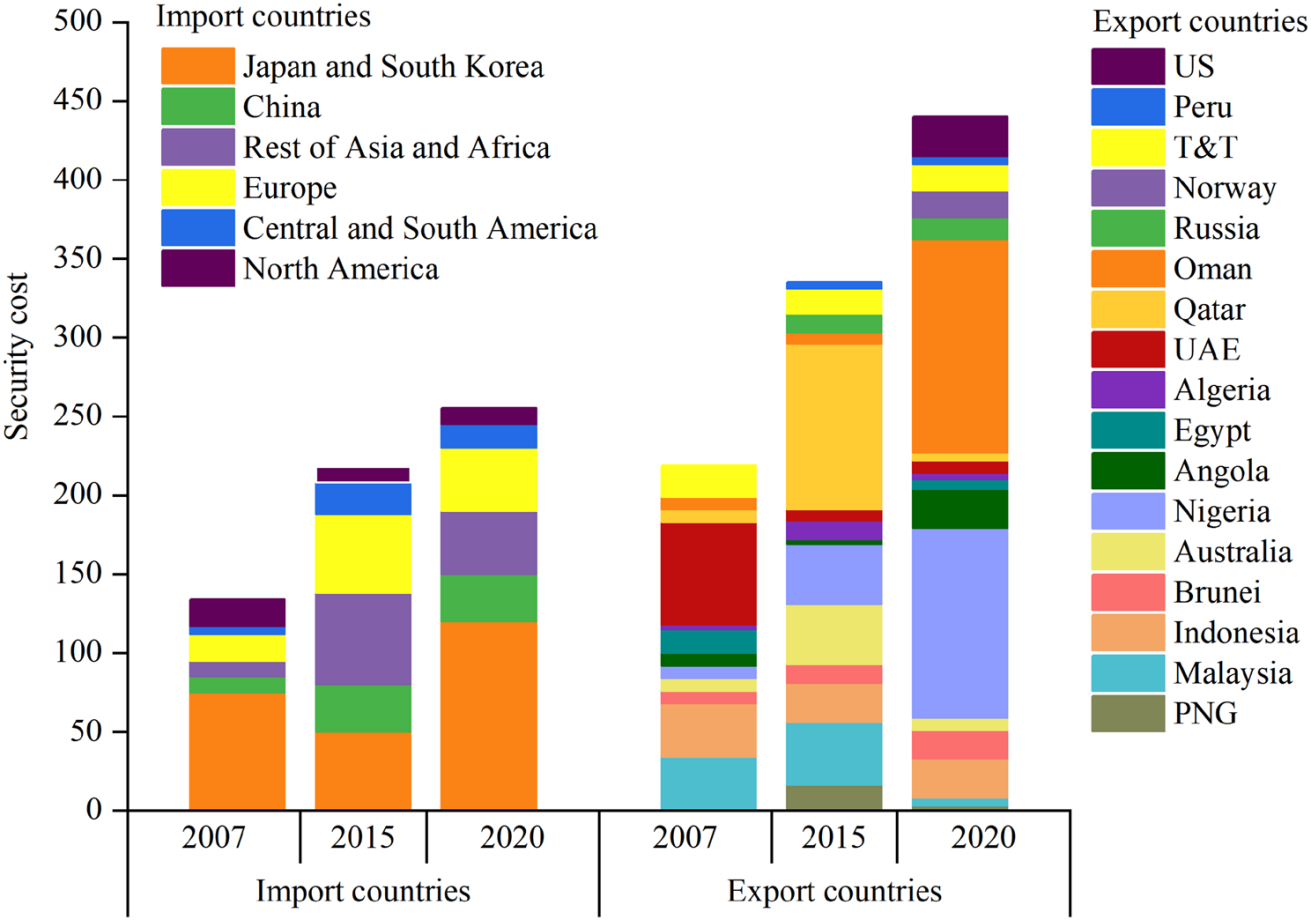
The export/import security costs of various LNG trading countries in different years.

LNG promotes the development of the international natural gas market from regional to global integration, and the cost of this process is shared by both supply and demand. This counterfactual result just reflects that the global natural gas market governance system is jointly shaped by both importers and exporters, and exporters share higher costs than importers. It is the flexibility of LNG that has transformed the natural gas market from supply to demand. Figure 3 shows the export/import security costs of various LNG trading countries in different years. For each LNG import market, the degree of scarcity faced by each import market is different. In terms of unit import security costs, North America, South America, and Japan and South Korea are the highest, followed by other markets. Because the demand for LNG in the American market is small and imports are only from neighboring countries, the degree of diversification of imports is low, so the cost of import security is higher than that of other markets. However, Europe, China, Japan and South Korea, and the Rest of Asia and Africa have a large import demand, high import maturity, and a wide range of import sources, and the scarcity faced by these countries is positively correlated with their import demand. In terms of total import security costs, the security costs in the Japanese and South Korean markets were the highest in the three years, which indicates that Japan and South Korea are more in short supply of LNG and the import costs are greater.

As far as LNG exporting countries are concerned, Qatar and Australia have the highest export security costs. Both countries have always been among the top exporters of LNG, and the export costs are also relatively high. In 2019, Australia's LNG export volume surpassed Qatar and became the largest export country. Australia is rich in natural gas resources, and many projects are still under construction. It is expected that future production will increase significantly, which will also alleviate the current shortage of LNG supply. Qatar, as one of the countries with the entire industrial chain of upper, middle, and lower reaches, has a high degree of flexibility and reliability in LNG export. Qatar is also improving infrastructure and equipment, reducing LNG production costs to increase its supply. Due to the shale gas revolution in the United States, other countries will also become the backbone of natural gas supply in the future.

**Figure 4:**
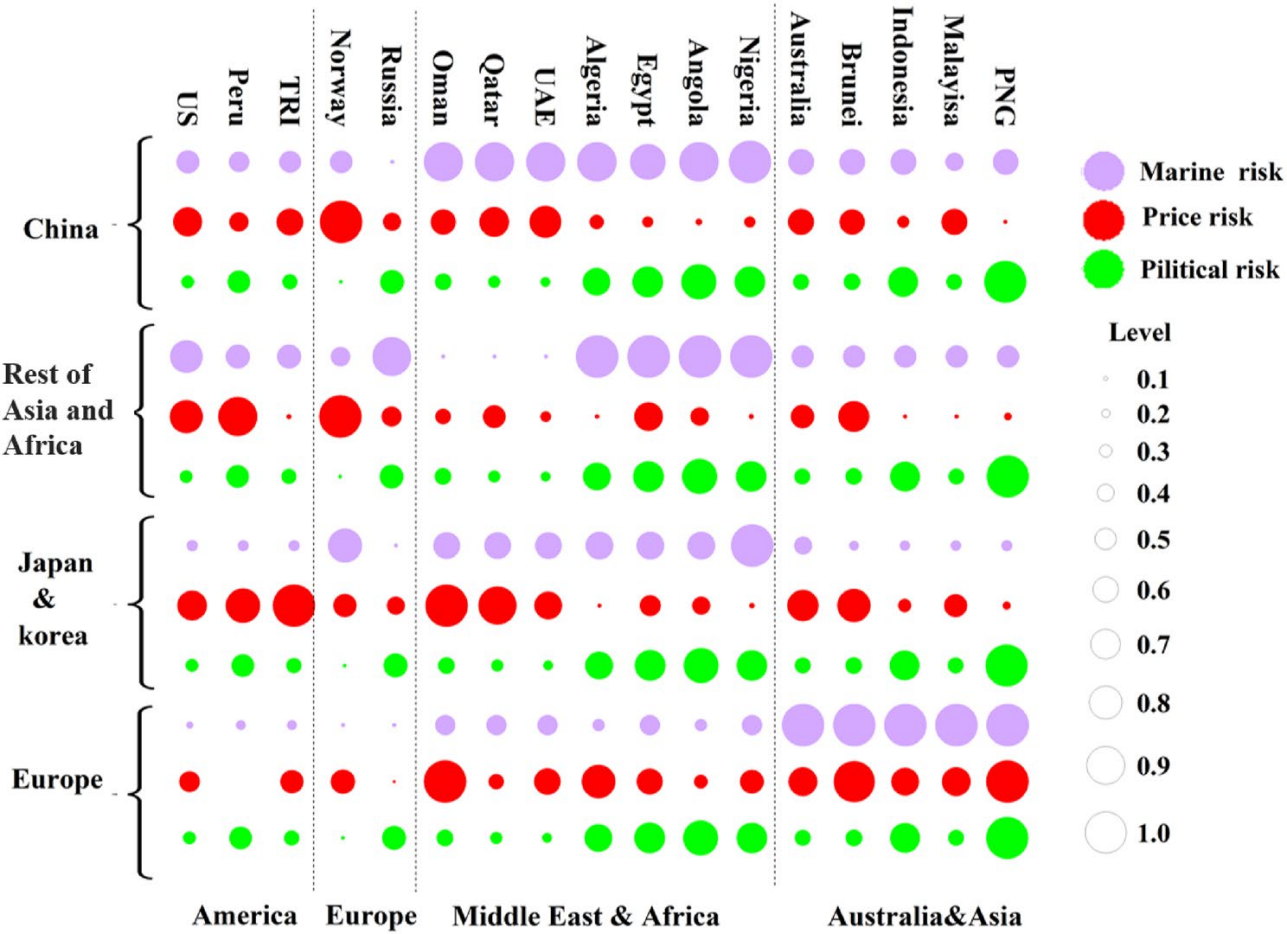
Risk identification of import sources in each LNG import market.

This section also analyzes the risk sources of the important LNG import market in 2020. Figure 4 shows the three types of risks in the four markets of China, Rest of Asia and Africa, Japan and South Korea, and Europe.

When there are political fluctuations or economic crises in the exporting country, it may cause the interruption of LNG supply and make the import market face a supply shortage of LNG. In 2020, countries with high national risks are mainly Peru, Egypt, Angola, and Papua New Guinea. Importing LNG from these countries will involve high-risk costs. In terms of price risk, due to the impact of regional price premiums and ocean freight, different markets have different import prices for various exporting countries. On the whole, China, Japan and South Korea’s import prices from the Middle East and Africa are much higher than those from other regions, while the Rest of Asian and African market’s import prices from the Americas are higher than other regions. Europe’s import prices from Southeast Asia are higher. As far as maritime risks are concerned, maritime risks are mainly affected by the distance of the sea and pirate attacks within the route. If the LNG importing country and exporting country are in the same region, the maritime risk is lower, otherwise, the risk is higher. Just talk about China, the source countries of high maritime risk come from the Middle East and North Africa. On the one hand, the sea distance is too long, and there are too many uncontrollable factors, which increase the risk; on the other hand, the Gulf of Aden and the Strait of Malacca are the necessary waterways for North Africa and the Middle East-China route, and frequent piracy also increases shipping risks. Imports from Southeast Asia, Australia, and the Americas have relatively low maritime risks.

Considering the three risks, the priority importing regions for China, Japan and South Korea are Southeast Asia and Australia. Importing LNG from these regions has low shipping and economic risks. However, the demand in the two markets is too high, the competition for LNG imports from the above-mentioned regional countries is high, and the LNG supply in this region cannot meet the import needs of the two markets. High supply despite high import risks is also an indispensable source of imports for the two markets. For the Rest of Asian and African market, the overall risk of importing LNG from the Middle East is low, and Southeast Asia is its second source of imports. Europe, the Americas, the Middle East, and North Africa are regions with low import risks. In summary, intra-regional imports are the preferred choice for reducing the risk of LNG imports. However, the increase in LNG demand cannot fully satisfy the LNG supply in the region. Although the risk of LNG imports from outside the region is higher, it is still a necessary source of supply.

### China’s import plan

The strong development of the LNG demand market and the increase in supply market capacity will increase the diversification and flexibility of the global trade market in the future, and the situation will become increasingly complex^32^. The degree of competition in the LNG market may continue to increase^33^.

By 2030, LNG supply growth is mainly concentrated in the United States, Australia, Qatar, Russia, Malaysia, and other countries^34^. With the investment in shale gas development, the United States is expected to reach 150 bcm of liquefaction capacity in 2030, becoming the world's largest exporter of LNG. For traditional exporters, the increase in LNG in the Middle East is mainly concentrated in Qatar^35^. All of its pending projects will be put into production before 2030, and the estimated production capacity will reach 148.2 bcm, which will still occupy a large market share. As Australia's LNG production boom has begun to turn, supplies will be limited for some time to come^36^. Although Russia's natural gas exports are mainly piped gas, it has been increasing its LNG capacity in recent years, and the commissioning of new LNG projects will bring its annual production capacity to 56 bcm per year by 2030. In Africa, Algeria and Egypt are affected by the source of raw gas and the LNG supply capacity is expected to remain at the current level in the future. By 2030, LNG demand is expected to double, with the main demand concentrated in Europe, China, and South Asia. The BP Energy Outlook expects China's LNG imports to double to 150 bcm around 2030, surpassing PNG imports. Europe and India will also see further expansion in LNG demand, while Japan and South Korea will maintain their current imports. Emerging LNG importers in Southeast Asia, such as Thailand, Singapore, and Bangladesh, will see a rapid increase in demand to reduce pollution. Supplementary Table a, b provide the estimated supply and demand of LNG trading countries in 2030^37^. Except for the countries mentioned in the table, other importing and exporting countries still retain the existing supply and demand.

**Figure 5:**
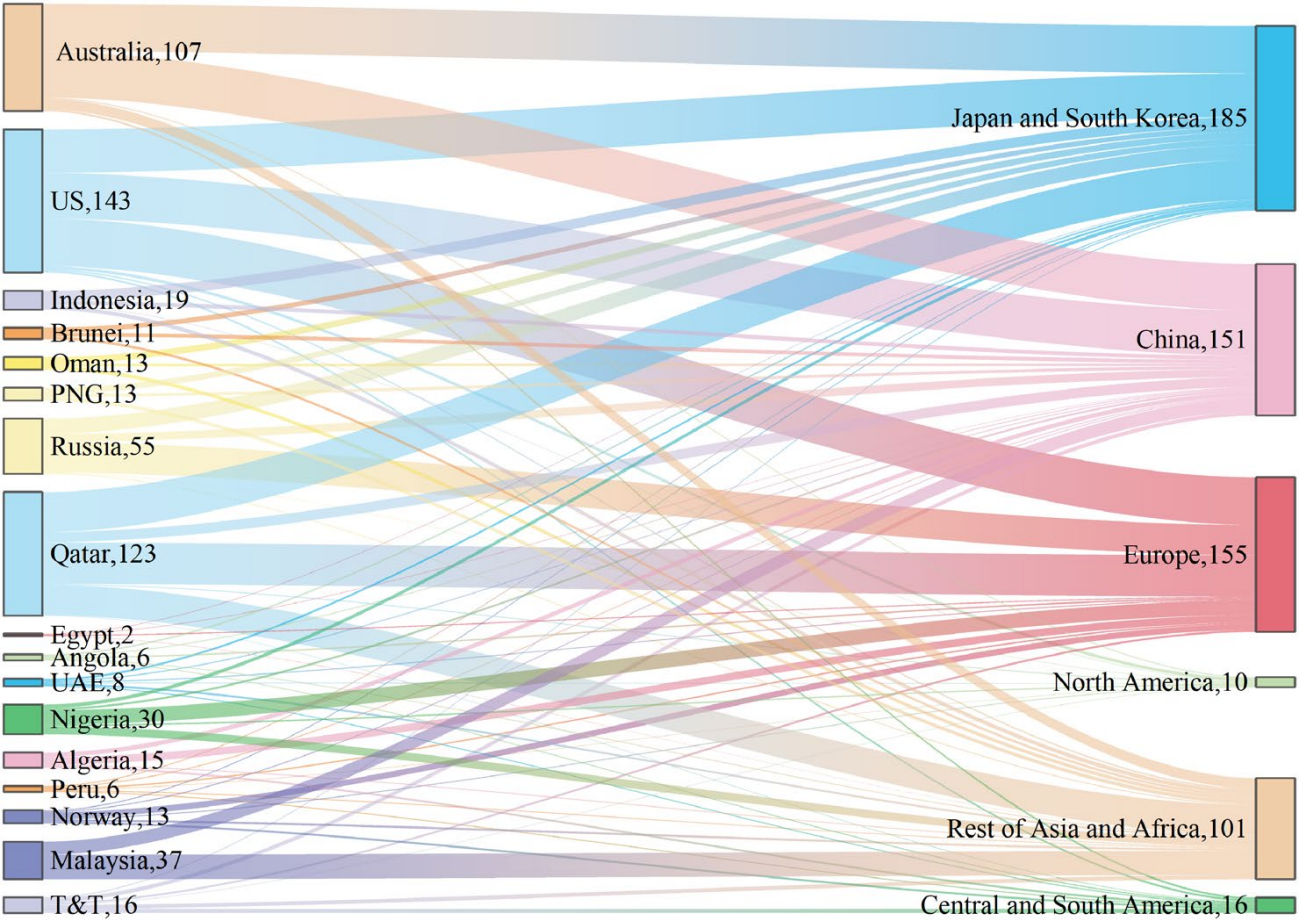
LNG trade strategy in 2030 at risk minimization (Figures represent the total volume of imports or exports in BCM).

In this section, we take the forecast of global LNG supply and demand in 2030 as the basic data to solve the model and get the game equilibrium result of global import and export trade when the LNG import risk is minimized in 2030, which is the corresponding import strategy. Figure 5 shows the LNG import portfolio transaction results in 2030. According to forecasts, in 2030, the United States, Australia, Qatar, Russia, and Malaysia will become the world's major LNG suppliers. The top five exporters will account for 75.36% of the LNG trade market, with the United States becoming the world's largest LNG exporter with a market share of about 25%. The growth of U.S. LNG exports mainly meets the LNG demand gap in Europe and China. The U.S. exports LNG to almost all import markets, which also makes its exports more diversified, ensuring its export security. From the prediction and simulation results, about 40% of the LNG import market demand in the Asia Pacific is supplied by four Southeast Asian countries and Australia, and the remaining demand mainly comes from the Middle East and the United States. Notably, the conflict between Russia and Ukraine has led Europe and the United States to tighten sanctions against Russia. The Financial Times reported that the EU plans to cut imports of pipeline gas from Russia by two-thirds and increase imports of LNG from the United States and other countries. Russia also plans to transfer the remaining natural gas to the Chinese market, so that China will reduce its dependence on LNG imports and import risks in the future.

**Figure 6:**
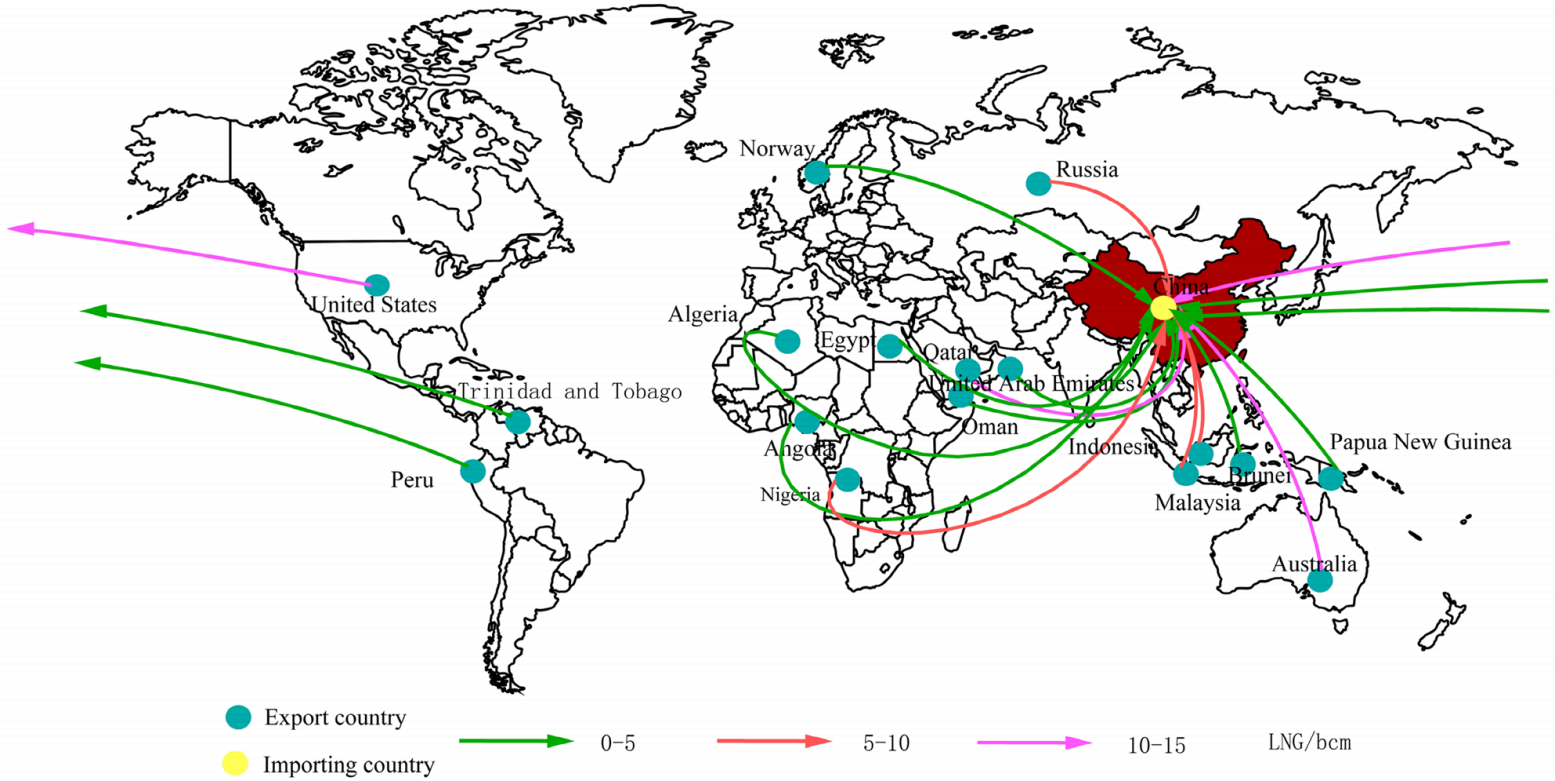
Simulation of China’s LNG import strategy in 2030.

It is estimated that by 2030, China's LNG import demand will reach 150 bcm, an increase of 38.3% over 2020. Figure 6 shows China's LNG import strategy for 2030 risk minimization. Qatar and Australia, which are major LNG exporters, also increased their supplies to China from 2020. However, from the perspective of reducing import risk and ensuring supply security, China cannot import too much LNG from these two countries to meet the demand gap. Our model finds that in 2030, China will import about 27% of its total LNG imports from the United States to meet its domestic demand. The reasons behind this are as follows: From the risk identification in the previous section, we can see that China's LNG import risks mainly come from Australia and the Middle East, and the import risks from the United States are smaller. Increased imports from the United States could reduce the risk of imports. In the optimal import strategy we obtained, Australia, the United States, and Qatar still occupy the largest market share in China. Meanwhile, to ensure the diversification of China's supply sources and reduce import risks, Southeast Asian and African markets are also China's import target countries. In addition, we use the model to simulate the sensitivity of LNG trade to changes in China's LNG demand, which could reshape LNG trade strategies through significant changes in its import trends. If China's LNG import demand increases to 170 bcm in 2030, due to import diversification restrictions, China cannot obtain surplus imports from the United States and Australia, and imports from Qatar have doubled.

## Conclusions and recommendations

From the perspective of the global LNG supply and demand pattern and maritime transportation network, this paper establishes a global LNG import multi-agent game model to evaluate China's LNG import risks and optimize import strategies in the future. Different from previous studies, this paper studies LNG import safety from the perspective of global LNG import market competition.

To verify the practicability of the proposed model, we conducted a risk assessment using global LNG trade data from previous years to analyze the import risks and costs of six energy import markets in several different years. For China, in the past, with the continuous increase of LNG imports, import risks have been increasing, especially shipping risks. China's LNG import risks mainly come from the Middle East and Africa. We find that imports from Southeast Asia, Australia, and The Americas have lower risks and can be used as priority import sources of LNG for China. In addition, intra-regional import is the preferred strategy for LNG to reduce import risk. However, LNG demand growth cannot be fully met by regional LNG supply, and imports from outside the region remain an essential source of supply despite the higher risk.

At the same time, with the future growth of China's LNG demand and the increasing degree of import competition in the LNG market, this paper simulates the game equilibrium result of the LNG world trade strategy in 2030 when the import risk is minimized and gives the optimal LNG import plan for China. In the future, to reduce import risks, the United States and Russia will become the new main sources of China's LNG imports, accounting for more than 50% of China's energy imports together with major exporting countries such as Australia and Qatar. At the same time, China should reduce its import dependence on the Middle East and Africa. The rest of the supply gap will be supplied by other long-term energy exporters, as import diversification strategies require.

Finally, we would like to offer some suggestions for further research directions in the future: extending the research period to a further distance such as 2060, introducing more potential LNG importers and exporters, and trading networks such as the Northeast Passage connecting Russia and East Asian markets; Consider both the global LNG market and the piped gas market; The model is further improved to facilitate risk sensitivity analysis.

## Supplementary Information

Supplementary Information.
