# Supplementary material for: China’s LNG import risk assessment based on the perspective of global governance
Source: Sci Rep. 2022 Sep 21;12:15754. doi: 10.1038/s41598-022-20090-1 (PMC9492653; doi:10.1038/s41598-022-20090-1)
Supplement: Supplementary file 1 — Supplementary Information. [file 41598_2022_20090_MOESM1_ESM.docx]

**Table a.** Forecast 2030 LNG supply and demand (bcm)

| supplier | 2030 Supply | demand market | Demand 2030 |
| --- | --- | --- | --- |
| USA | 150 | North America | 9.6 |
| Russia | 56 | Central and South America | 14.5 |
| Qatar | 148.2 | Europe | 147 |
| Australia | 109 | Rest of Asia and Africa | 100 |
| Malaysia | 43.9 | China | 150 |
|  |  | Japan and South Korea | 173.5 |

**Table b.** Trading price ($/m^3^) and ICRG index

|  | North America | Central and South America | Europe | China | Japan and South Korea | Rest of Asia and Africa | ICRG index |
| --- | --- | --- | --- | --- | --- | --- | --- |
| USA | 0.7214 | 0.3044 | 0.3174 | 0.4214 | 0.4112 | 0.4312 | 84.3 |
| Peru | 0.3647 | 0.2926 | 0.2088 | 0.4981 | 0.4277 | 0.4524 | 77.8 |
| T&T | 0.3334 | 0.3085 | 0.3304 | 0.3334 | 0.4561 | 0.3246 | 82.8 |
| Norway | 0.1527 | 0.1527 | 0.3356 | 0.5239 | 0.3884 | 0.4662 | 93.3 |
| Russia | 0.3334 | 0.3334 | 0.2238 | 0.3334 | 0.3698 | 0.3815 | 77.0 |
| Oman | 0.4305 | 0.4305 | 0.4305 | 0.4305 | 0.4554 | 0.3650 | 81.8 |
| Qatar | 0.1527 | 0.2995 | 0.2890 | 0.4478 | 0.4409 | 0.3919 | 84.8 |
| UAE | 0.3474 | 0.4046 | 0.3474 | 0.4045 | 0.4045 | 0.3474 | 86.3 |
| Algeria | 0.5316 | 0.2734 | 0.3830 | 0.2987 | 0.3064 | 0.3064 | 74.5 |
| Angola | 0.5316 | 0.2878 | 0.2807 | 0.3262 | 0.3698 | 0.3759 | 72.3 |
| Eygpt | 0.3262 | 0.3262 | 0.3440 | 0.3808 | 0.3808 | 0.4147 | 69.5 |
| Nigeria | 0.3262 | 0.3262 | 0.3334 | 0.3262 | 0.3262 | 0.3262 | 72.5 |
| Australia | 0.3592 | 0.3592 | 0.3592 | 0.3592 | 0.4161 | 0.3952 | 82.3 |
| Brunei | 0.4133 | 0.4133 | 0.4233 | 0.4133 | 0.4233 | 0.4233 | 81.8 |
| Indonesia | 0.3896 | 0.3896 | 0.3538 | 0.3896 | 0.3538 | 0.3065 | 73.0 |
| Malaysia | 0.3472 | 0.3472 | 0.3592 | 0.3472 | 0.3874 | 0.3093 | 82.3 |
| PNG | 0.4301 | 0.4301 | 0.4301 | 0.4301 | 0.3349 | 0.3349 | 64.8 |
